# A Diabetes Self-Management Program: 12-Month Outcome Sustainability From a Nonreinforced Pragmatic Trial

**DOI:** 10.2196/jmir.6484

**Published:** 2016-12-15

**Authors:** Kate Lorig, Philip L Ritter, Ralph M Turner, Kathleen English, Diana D Laurent, Jay Greenberg

**Affiliations:** ^1^ Stanford Patient Education Research Center Medicine Stanford University Palo Alto, CA United States; ^2^ HealthCore Wilmington, DE United States; ^3^ Anthem Inc Indianapolis, IN United States; ^4^ NCOA Services, LLC Arlington, VA United States

**Keywords:** patient education, self-management, type 2 diabetes

## Abstract

**Background:**

Diabetes self-management education has been shown to be effective in controlled trials. The 6-week Better Choices, Better Health-Diabetes (BCBH-D) self-management program was also associated with an improvement in health outcomes in a 6-month translation study.

**Objective:**

The objective of this study was to determine whether a national translation of the BCBH-D self-management program, offered both Web-based and face-to-face, was associated with improvements in health outcomes (including HbA1c) and health behaviors (including recommended medical tests) 1 year after intervention

**Methods:**

Web-based programs were administered nationally, whereas face-to-face workshops took place in Atlanta, Indianapolis, and St Louis. Self-report questionnaires were either Web-based or administered by mail, at baseline and 1 year, and collected health and health-behavior measures. HbA1c blood samples were collected via mailed kits. A previous 6-month study found statistically significant improvements in 13 of 14 outcome measures, including HbA1c. For this study, paired *t* test compared baseline with 1-year outcomes. Subgroup analyses determined whether participants with specific conditions improved (high HbA1c, depression, hypoglycemia, nonadherence to medication, no aerobic exercise). The percentage of participants with improvements in effect size of at least 0.4 in at least 1 of the 5 measures was calculated.

**Results:**

A total of 857 participants with 1-year data (69.7% of baseline participants) demonstrated statistically significant 1-year improvements in 13 of 15 outcome measures; 79.9% (685/857) of participants showed improvements in effect size of 0.4 or greater in at least 1 of the 5 criterial measures.

**Conclusions:**

Participants had small but significant benefits in multiple measures. Improvements previously noted at 6 months were maintained or amplified at 1 year.

## Introduction

### Background

Although Healthy People 2020 recommends diabetes education, less than 7% of people with diabetes report receiving formal diabetes education in the year following diagnosis [1]. Diabetes education may also help with achieving many of the Healthcare Effectiveness Data and Information Set (HEDIS) measures [2]. Diabetes self-management education aims to increase healthful behaviors while reducing HbA1c. In a recently published paper, we reported on the 6-month outcomes for a diabetes self-management intervention offered both face-to-face and Web-based [3]. Study participants improved healthful behaviors, medication adherence, hypoglycemia, and HbA1c. More than 70% of study participants made a positive improvement (effect size of 0.4 or greater) in 1 or more of 5 outcome variables (HbA1c, frequency of hypoglycemia, medication adherence, completing recommended screenings, and exercise). In this paper, we report on the 1-year outcomes for the same study.

### Hypotheses

The study aimed at testing the following hypotheses:

1. Improvements in HbA1c, health indicators (hypoglycemic symptoms and depression), and health behaviors (exercise, medication adherence, receiving recommended tests) between baseline (preintervention) and 1 year.

2. Changes would meet or exceed Healthy People diabetes recommendation for percentage of population with HbA1c above 9% and below 7% and percentage of population receiving microalbumin, foot, and eye examinations.

3. Effectiveness would be independent of the mode of delivery.

4. Both older (65 plus) and younger participants would benefit.

5. Participants with baseline HbA1c ≥9.0, Patient Health Questioinaire (PHQ-8) depression ≥10.0, 2 or more hypoglycemia symptoms, medication nonadherence, or no aerobic exercise would have clinically significant improvements in these variables.

6. Moderate effect size (0.4) improvements would be found for the majority of participants in reducing 1 of more of the aforementioned variables.

## Methods

### Intervention

Better Choices, Better Health-Diabetes (BCBH-D) was developed for people with type 2 diabetes. Both versions of the program (face-to-face and Web-based) meet the American Association of Diabetes Educators (AADE) standards for diabetes self-management and support [4], have been shown to be effective in previous randomized trials [5,6], have the same content, and are designed to enhance self-efficacy [7]. Content includes healthy eating, exercise, understanding glucose monitoring, communicating with family, friends, and the health care system, hypoglycemia, depression, difficult emotions, sick days, medication management, problem solving, decision making, and action planning. Both are 6 weeks, have 2 peer facilitators, and have standardized facilitator training. Neither program had any reinforcement beyond the initial intervention. Both have been described in detail elsewhere [3]. The study was approved by the Stanford and New England institutional review boards and has 5 collaborators, Anthem Inc, the National Council on Aging, Stanford School of Medicine, the National Council of Young Men’s Christian Association of the United States of America, and OASIS Health.

### Recruitment

Because this was a pragmatic trial designed to be offered in real-world settings, there were few inclusion (have type 2 diabetes and be covered by an Anthem-affiliated health plan) or exclusion (currently pregnant or in chemotherapy or radiation treatment for cancer) criteria. Unlike most efficacy trials, symptom severity was not an inclusion criterion.

Web-based participants were recruited in 2013 and 2014 by email from their employers or emails or phone calls from an Anthem plan. Both commercial and Medicare-Advantage participants were eligible. Potential study participants went to the recruitment website, completed screening, and completed an informed consent and baseline questionnaire.

Face-to-face participants were recruited through mailings, flyers in workplaces or physicians’ offices, case managers, and automated telephone calls. Face-to-face programs were available in Atlanta, Indianapolis, and St. Louis. A small percentage of the community participants were not covered by Anthem plans. All other screening criteria were the same for Web-based and face-to-face participants.

### Data Collection

Data were collected using self-report, validated questionnaires at baseline and 12 months. We asked participants to furnish a sample of blood, although this was not required for program or study participation; nor were participants disqualified if they failed to return their samples. Consenting participants were sent HbA1c test kits. These were returned to investigators, bar-coded to avoid disclosing PHI, and then sent to CoreMedica, a Clinical Laboratory Improvement Amendments (CLIA) certified lab [8]. Participants and their physicians were sent results. Because CoreMedica recalibrated its measurements in June 2014 increasing all values by roughly 0.4, all measures prior to June were adjusted upward by 0.4.

### Measures

Measures were chosen to be of interest to patients, providers, and the health care system. Demographic variables included age, gender, race, ethnicity, education, and marital status. Participant also reported other diseases. Previously validated outcome measures are described in detail elsewhere [3,9,10].

Health indicators included self-rated health, which is a single item from the National Health Examination Survey [11], the PHQ-8 depression scale [12], the Illness Intrusiveness Scale, which measures role function [13], and the hypoglycemic symptoms scale [14]. Fatigue and sleep are each single-item visual numeric scales [15]. Behavior indicators included communication with physicians, a scale asking how often patients ask questions and discuss problems with their health care provider [10], minutes of aerobic exercise per week [10], and the Morisky Medication Adherence scale [16]. We also asked participants if they had eye, foot, cholesterol, and kidney examinations in the last year or, on follow-up questionnaires, in the last 6 months.

### Data Analysis

Pragmatic studies, because of study-population heterogeneity, present unique methodological challenges. In many diabetes studies, subjects are chosen, for example, because of high HbA1c or depression. In this study, no such screening occurred, resulting in greater heterogeneity for the key outcome variables. Not all participants have the same problems, and some have no problems. Consequently, we conducted several types of analyses. The first was a descriptive analysis of the participants and their engagement with the program. The second, or classic, set of analyses determined the 12-month changes and significance for the population as a whole. The third, or subset, analyses examined only those who demonstrated problems in specific variables of interest, for example, high HbA1c or low adherence to taking medications. Having a problem was decided either by a standard criteria such as 10 or above on a PHQ-8 being highly suggestive of depression [12] or by specific scores on the adherence and hypoglycemia scales suggested by the authors and previous publications. For exercise, we chose those not exercising at baseline, and for HbA1c, we chose 9, as this is the level discussed in the Healthy People 2020 goals [17]. A fourth analysis sought to reconcile the second and third analyses by examining the percentage of the total population who achieved a moderate benefit (0.4 effect size) in at least 1 of the 5 variables of interest.

To help determine the likelihood of bias caused by attrition, we compared baseline scores of participants who failed to complete with those who completed 12-month questionnaires.

Univariate statistics describe demographic and engagement characteristics. Independent-sample *t* tests compare demographic and baseline outcome variables between those who failed to complete 12-month follow-up questionnaires and those who completed them. Paired *t* tests examined changes between baseline and 12 months and if these differed significantly from a null hypothesis of zero change (hypothesis 1).

For those who had had no examinations (eye, foot, cholesterol, or kidney) in the year prior to entry, we calculated the percentage that had examinations in the 12 months following baseline.

To compare effectiveness by mode of delivery, we used independent- sample *t* tests to compare change scores between Web-based and face-to-face participants (hypothesis 3). Similarly, we compared 1-year changes for older (65 plus) and younger participants (hypothesis 4).

Subgroup analyses were conducted for participants with specific conditions as described above: HbA1c above 9; clinical depression (PHQ-8 of 10 or above [12]); at least two symptoms of hypoglycemia; low medication adherence; and no exercise at baseline. For each measure, we report the mean change of the group and the percentage that no longer had the negative indication (hypothesis 5). In addition, to examine the possibility that results were due to regressions to the mean, we calculated the change scores for the subsets of the sample that did not meet each of the 5 negative criteria.

To determine the proportion of all participants who benefited on at least 1 of the 5 indicators, we calculated the percentage who improved by an effect size of at least 0.4 (hypothesis 6). We examined the relationship between the number of criterial indicators and the number of improvements using Pearson correlations.

## Results

### Participants

**Figure 1:**
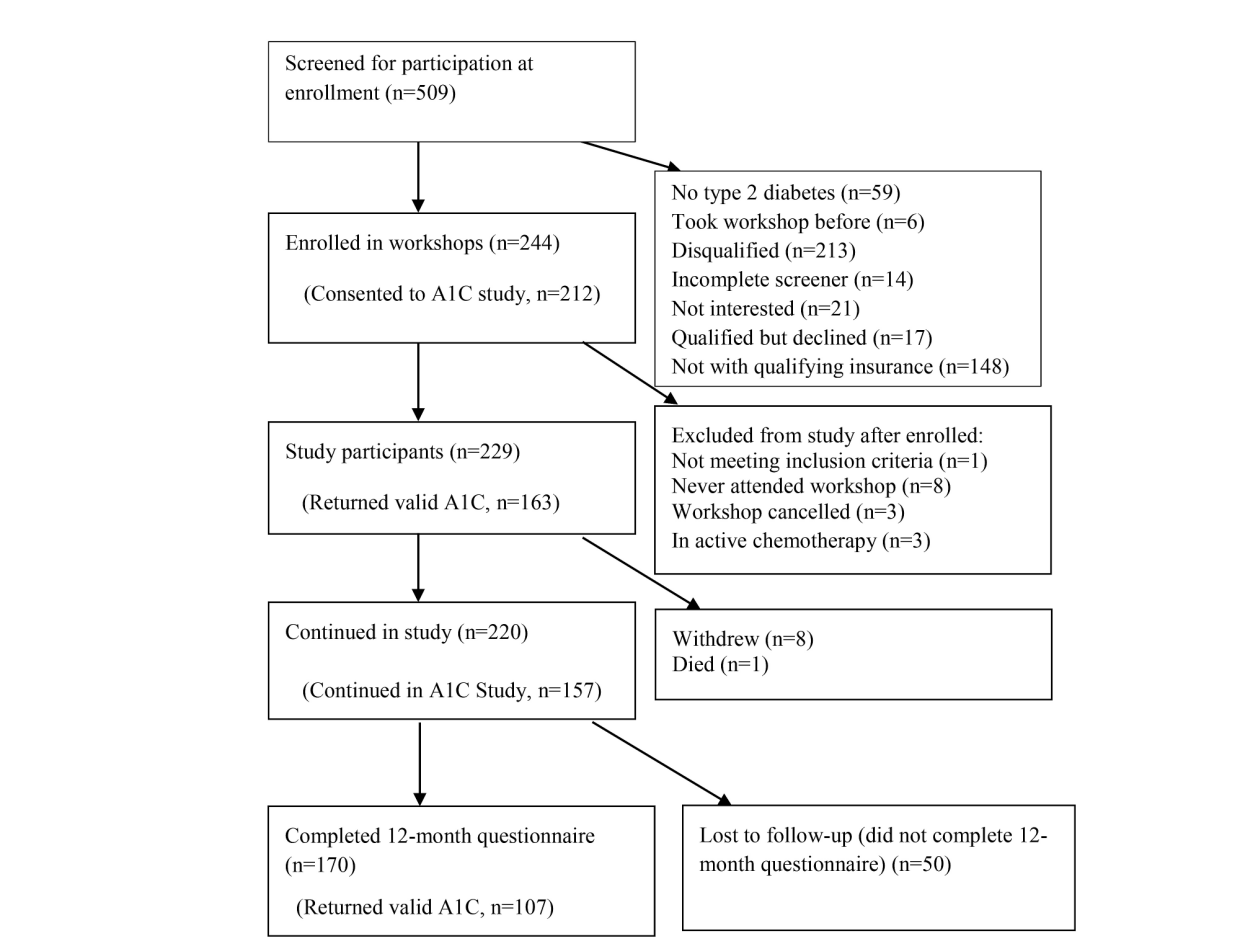
Face-to-face workshops CONSORT flowchart.

**Figure 2:**
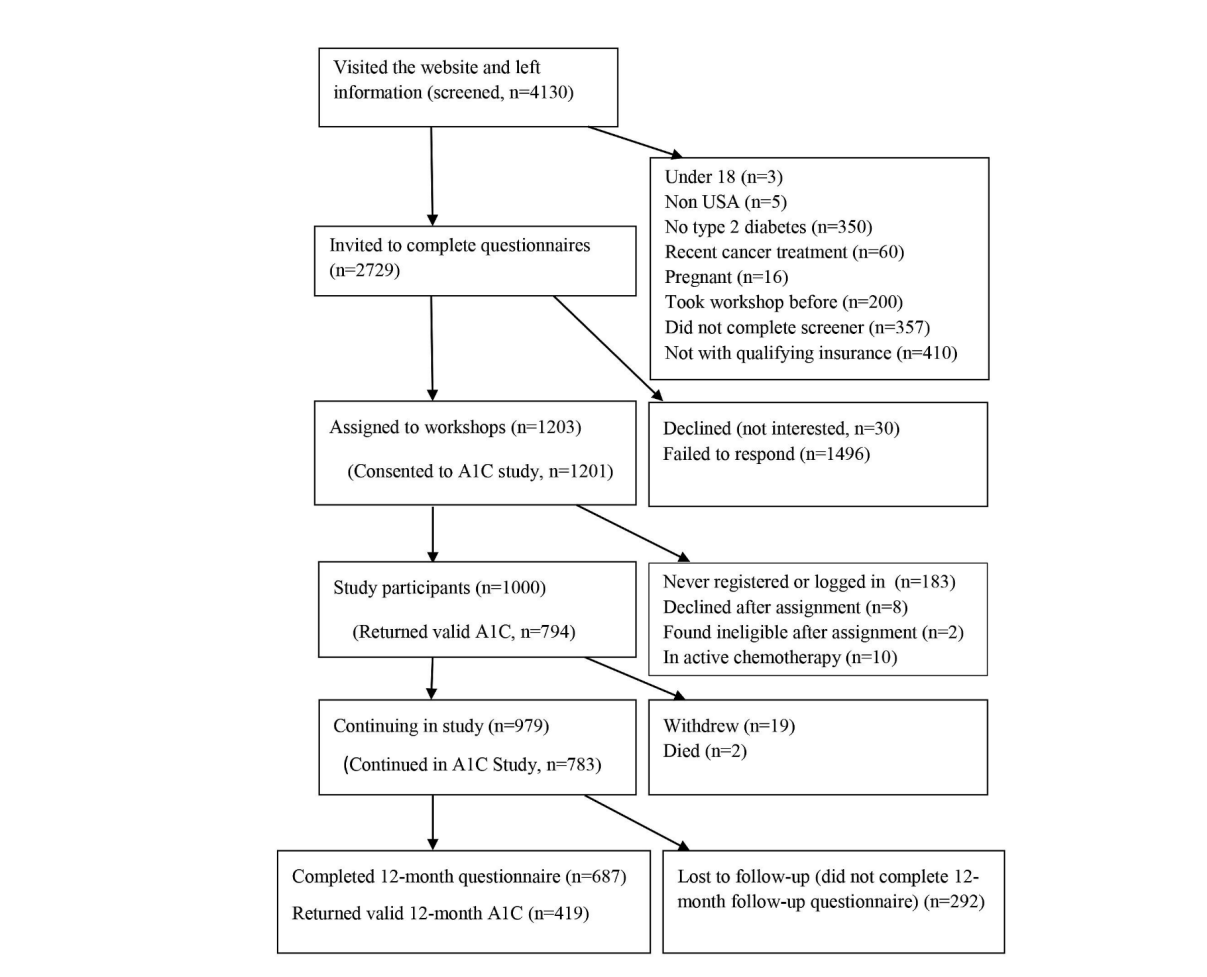
Web-based CONSORT flowchart.

A total of 4639 potential participants (509 face-to-face and 4130 Web-based) left contact information. All were invited to fill out a screener and, if eligible, complete consent and the baseline questionnaire. People only became study participants when they attended the first workshop or logged on to the first session. Of these, 1229 (229 face-to-face and 1000 Web-based) completed the process, were eligible, and became study participants. The majority of those not completing the process did not have Anthem-affiliated plans or failed to complete the screener, consent, or questionnaire (Figures 1 and 2). Of those starting the program, 687 and 170 (69.7%) completed 12-month questionnaires. About 80% (957/1229) of the participants successfully completed a baseline HbA1c. Of these, 55.0% (526/957) completed 12-month HbA1cs.

Table 1 shows demographic characteristics and baseline outcome scores. Relative to the face-to-face cohort, the Web-based participants were more likely to be male, more likely to be married, and less likely to be minority. They also were younger. All differences were statistically significant. There was 1 statistically significant difference between the 2 groups among baseline outcome measures. The face-to-face participants had better initial communication with their physicians (*P=*.03).

**Table 1:** Baseline characteristics of participants with 12-month data.

Variable	Face-to-face (n=170)	Web-based (n=687)	All (N=857)
Male, n (%)	47 (27.6)	238 (34.6)	285 (33.3)
Education in years, mean (SD, range)	15.2 (2.91, 8-23)	15.6 (2.78, 10-23)	15.5 (2.81, 8-23)
Married, %	89 (52.3)	518 (75.5)	607 (70.9)
Non-Hispanic white, %	114 (67.1)	524 (83.5)	638 (74.4)
Black, %	49 (28.8)	57 (8.3)	106 (12.4)
Hispanic, %	3 (1.8)	60 (8.7)	63 (7.4)
Age in years, mean (SD, range)	65.6 (9.95, 28-95)	55.8 (8.62, 26-91)	57.7 (9.71, 26-95)
Number of other chronic conditions, mean (SD, range)	1.65 (1.37, 0-8)	1.40 (1.15, 0-6)	1.45 (1.19, 0-8)
Medicare, %	97 (57.6)	48 (7.0)	145 (16.9)
Private insurance, %	124 (72.9)	687 (100)	811 (94.5)
HbA1c ↓^a^, n=732, mean (SD, range)	7.79 (1.55, 5-15.2)	8.02 (1.28, 5-12.1)	8.04 (1.44, 5-14.8)
PHQ-8 depression ↓, mean (SD, range)	5.91 (4.99, 0-23)	5.94 (4.87, 0-23)	5.92 (4.96, 0-23)
General health ↓, mean (SD, range)	2.84 (0.760, 1-5)	2.87 (0.812, 1-5)	2.87 (0.770, 0-5)
Illness intrusiveness ↓, mean (SD, range)	2.63 (1.25, 1-7)	2.83 (1.11, 1-6.07)	2.80 (1.22, 1-7)
Hypoglycemic symptoms ↓, mean (SD, range)	1.35 (1.42, 0-6)	1.39 (1.40, 0-6)	1.39 (1.42, 0-6)
Fatigue ↓, mean (SD, range)	4.66 (2.31, 0-10)	4.86 (2.31, 0.10)	4.83 (2.31, 1-10)
Sleep ↓, mean (SD, range)	3.82 (2.89, 0-10)	3.92 (2.97, 0-10)	3.91 (2.91, 1-10)
Aerobic exercise (min/week) ↑, mean (SD, range)	95.3 (98.5, 0-555)	81.4 (104, 0-720)	84.0 (99.6, 0-720)
Stretching/range of motion (min/week) ↑, mean (SD, range)	34.0 (43.9, 0-180)	26.1 (49.8, 0-180)	27.6 (45.1, 0-180)
Communication with MD ↑, mean (SD, range)	2.78 (1.15, 0-5)	2.57 (1.23, 0-5)	2.61 (1.08, 0-5)
Medication adherence ↓, mean (SD, range)	0.971 (1.11, 0-4)	1.12 (1.02, 0-4)	1.09 (1.17, 0-4)
Proportion eye exam, last 12 months^b^, %	131 (77.1)	533 (77.6)	664 (77.5)
Proportion foot exam, last 12 months^b^, %	126 (74.1)	469 (69.7)	605 (70.5)
Proportion cholesterol exam, last 12 months^b^, %	154 (90.6)	639 (93.0)	793 (92.5)
Proportion kidney exam, last 12 months^b^, %	125 (73.5)	534 (77.7)	659 (76.8)

^a^Because of a lab recalibration changing HbA1c measurement, HbA1c prior to June 2014 was adjusted by adding 0.4. ↑ Indicates that a higher score is desirable, and ↓ that a lower score is desirable.

^b^“Don’t know” set to no exam.

### Program Participation and Engagement

Participants took part in 1 of 50 face-to-face workshops or 49 Web-based workshops. Face-to-face workshops had 3-16 study participants attending a mean of 4.7 of 6 sessions (SD 1.5). Web-based workshops had 5-28 participants. Both modes also had nonstudy participants.

Web-based participants logged in a mean of 4.8 sessions (SD 1.5, range 1-6). They averaged 4.36 action plans (SD 3.9, range 0-6), 5.25 posts to discussion boards (3.9, 0-22), 10.4 replies to posts (20.1, 0-228), and 157.0 visits to workshop Web pages (62.4, 1-209).

### Those Failing to Complete 12-Month Questionnaires

Of 1229 participants, 69.7% (857/1229) completed the 12-month questionnaire. The face-to-face participants (74.2%, 170/229) were more likely to complete compared to Web-based participants (68.7%, 687/1000). Outside of optional HbA1c, no variable had more than 1.7% missing data among 12-month completers. Nine-hundred and fifty-three consented to HbA1c testing and successfully completed baseline tests. Twelve-month HbA1c tests were successfully completed by 526 participants (55.0% (526/957) of those consenting and furnishing baseline HbA1c samples).

We compared baseline values of those not completing 12-month questionnaire and completers. Among demographic variables, noncompleters tended to be younger (mean 55.1 vs 57.8 years old, *P*<.001) and slightly less educated (14.9 vs 15.5 years of education, *P*<.001). There were no significant differences by gender, marital status, or ethnicity.

Among 15 outcome variables, 4 had statistically significance differences at baseline comparing completers and noncompleters. Also, 12-month noncompleters reported higher baseline HbA1c (8.4% vs 8.1% **,** *P=*.007), lower general health (3.04 vs 2.87 on a 1-5 scale, *P*<.001), less aerobic exercise (65.3 vs 84.0 minutes/week, *P=*.002), and less likely to have had an eye exam in the last 12 months (79.7% vs 77.5%, *P*=.005).

### Changes From Baseline

Table 2 shows the mean changes from baseline to 12 months (hypotheses 1). Seven of 7 health indicators (including HbA1c) and 7 of 8 health behaviors had statistically significant improvements between baseline and 12 months.

If we apply a Bonferroni correction and use .003 as the level of significance, 12 of 15 outcomes remain statistically significant.

**Table 2:** Baseline to 12-month scores, 12-month participants (N=857).

Variable^a^	Baseline mean (SD)	12-month mean (SD)	Baseline to 12-Month		*P* > |t| Baseline to 12 months
			Mean change	95% CI	
HbA1c ↓ ^b^ (n=526)	8.04 (1.44)	7.60 (1.55)	–0.447	–0.559 to –0.348	<.001
PHQ-8 depression (0-24) ↓	5.92 (4.96)	4.89 (4.87)	–1.02	–1.32 to –0.753	<.001
General health (0-5) ↓	2.87 (0.770)	2.77 (0.768)	–0.097	–0.140 to –0.048	<.001
Illness intrusiveness (1-7) ↓	2.79 (1.22)	2.65 (1.29)	–0.133	–0.214 to –0.065	<.001
Hypoglycemic symptoms (0-7) ↓	1.39 (1.42)	1.13 (1.29)	–0.260	–0.352 to –0.171	<.001
Fatigue (1-10) ↓	4.82 (2.31)	4.28 (2.52)	–0.541	–0.707 to –0.385	<.001
Sleep (1-10) ↓	3.91 (2.91)	3.68 (2.82)	–0.223	–0.427 to –0.040	.02
Aerobic exercise (min/week) ↑	84.0 (99.6)	101 (102)	16.7	9.91 to 23.4	<.001
Stretching or range of motion (min/week) ↑	27.6 (45.1)	34.0 (47.7)	6.28	2.83 to 9.52	<.001
Communication with MD (0-5) ↑	2.61 (1.08)	2.87 (1.20)	0.255	0.185 to 0.322	<.001
Medication adherence (0-4) ↓	1.09 (1.17)	0.917 (1.07)	–0.180	–0.240 to –0.103	<.001
Proportion eye exam, last 12 months (0,1) ↑^c^	0.778	0.849	0.070	0.035 to 0.105	<.001
Proportion foot exam, last 12 months (0,1) ↑^c^	0.710	0.799	0.090	0.056 to 0.123	<.001
Proportion cholesterol exam, last 12 months (0,1) ↑^c^	0.932	0.947	0.014	–0.007 to 0.036	.19
Proportion kidney exam, last 12 months (0,1) ↑^c^	0.776	0.885	0.109	0.077 to 0.141	<.001

^a^Possible range given in parentheses after variable name.

^b^Because of a lab recalibration changing HbA1c measurement, HbA1c prior to June 2014 was adjusted by adding 0.4. ↑ Indicates that a higher score is desirable, and ↓ that a lower score is desirable.

^c^“Don’t know” set to no exam, change scores for those in both 6- and 12-month follow-ups.

#### 12-Month Intent-to-Treat Analyses

For “intent-to-treat” analyses, we followed the standard practice of assuming no change for 12-month change scores for those who failed to complete 12-month questionnaires. The intent-to-treat analyses of changes resulted in no differences from the *P* values shown in Table 2.

#### HbA1c Changes

About 80% (957/1229) of participants supplied a valid HbA1c sample at baseline, and of these, 54.9% (526/957) supplied a 12-month HbA1c. There were no significant differences in any of the other baseline outcome measures for those who supplied an HbA1c sample versus those who did not. Participants who supplied an HbA1c sample had a slightly higher mean age (57 vs 55, *P*=.04), and were more likely to be of non-Hispanic white ethnicity (82.3% vs 68.5%, *P*<.001).

Because the lab that did the HbA1c testing recalibrated its measurements in June 2014 increasing all values by roughly 0.4%, all measures prior to June (some baseline and some 6-month) were adjusted upward by 0.4. If we remove all those with adjusted HbA1c at baseline (all whose baseline was before June 2014), the 12-month improvement in HbA1c remains almost the same (–0.454 vs –0.456). This suggests that our adjustment was appropriate.

#### Changes in Screening Tests (Hypotheses 1 and 2)

Table 3 gives the proportion of the participants who had each of 4 recommended tests in the year before baseline and the year between baseline and 12-month questionnaire. The proportion significantly increased for foot, eye, and kidney tests (*P*<.001). The proportion having cholesterol tests increased but not significantly, as it was already at a high level (93.2%) at baseline. For those not having a recommended test during the preintervention year, foot, eye, cholesterol, and kidney testing increased significantly. The total number of tests in the previous year also increased significantly from a mean of 3.2 (out of 4) to 3.5 (*P*<.001).

**Table 3:** Proportion receiving recommended examinations (N=768).

Type of exam^a^	Percent of those who had exam in 12 months prior to baseline, % (n)	Percent of those who had exam in 12 months between baseline and 1 year^b^, % (n, *P* value)	Percent of those who had no exams 12 months prior to baseline against those who had exam in 12 months after baseline^c^, % (n, *P* value)
Foot	71.0 (545)	80.0 (614, *P*<.001)	55.6 (124/223, *P*<.001)
Eye	77.9 (598)	84.9 (652, *P*<.001)	71.2 (121/170, *P*<.001)
Cholesterol	93.2 (716)	94.7 (742, *P*=.19)	76.9 (40/52, *P*<.001)
Kidney albumin	77.6 (592)	88.5 (680, *P*<.001)	72.7 (125/172, *P*<.001)

^a^Cases are those who had baseline, 6-month, and 12-month questionnaires.

^b^For *P* values in column 3, *t* tests were used to compare the proportion of those who had had the exam prior to baseline with the proportion of those who had had the exam between baseline and 12 months.

^c^For *P* values in column 4, chi-square test was used.

#### Web-Based Versus Face-to-Face Participants (Hypothesis 3)

Using *t* tests, only 4 1-year changes were found to be significantly different between Web-based and face-to-face participants: general health, sleep problems, stress, and medication adherence. These were further examined using general linear models controlling for the baseline values and for demographic covariates (age, gender, education, and whether minority). Differences in changes in general health were no longer significant after covariate adjustment. Web-based participants had greater improvements in medication adherence (*P=*.02), while face-to-face participants had greater improvements in sleep (*P=*.03) and stress (*P=*.002).

#### Older Versus Younger Participants (Hypothesis 4)

We segmented participants into those 65 and older (Medicare eligible, n=166) and those less than 65 years of age (n=691). There were 2 statistically significant differences in 12-month outcomes. The older group had significantly less increase in stretching and strengthening exercise (*P=*.01), and in general health (*P=*.02). For all other outcomes, change scores were similar. In particular, those over 65 decreased HbA1c by 0.43 while those under 65 similarly decreased HbA1c by 0.45.

#### Analyses of Participants With Specific Baseline Conditions (Hypothesis 5)

Because of sample heterogeneity, we examined change scores for specific outcomes for only those who had at least one of 5 criterial problems at baseline (high HbA1c, depression, hypoglycemia, low medication adherence, or no aerobic exercise).

##### High HbA1c

About 43% (524/1229) of the total sample had 12-month HbA1c results, and of those with 12-month HbA1c scores, 22.5% (118/524) had a baseline HbA1c of 9.0% or greater. By 12 months, approximately a third of these (30.3%, 36/119) had an HbA1c below 9. The mean decrease in HbA1c for those starting at above 9 was –1.27. Of those below 9 at baseline (n=407), the mean decrease was –0.206.

At baseline, 22.3% (117/524) had an HbA1c less than 7.0. By 12 months, 36% (189/524) of the participants were below 7, an increase of 61.5% (72/117). The 407 12-month participants with HbA1c of 7.0% or more at baseline had a mean 12-month decrease in HbA1c of 0.599, while those less than 7 at baseline (n=117) had a slight increase of 0.084.

##### No Aerobic Exercise

Participants who reported no aerobic exercise at baseline (n=185) had a mean increase of 46.0 minutes/week at 12 months. Those who reported some aerobic exercise had a mean increase of 8.6 minutes. The American Diabetes Association recommends that people with diabetes should have 150 minutes per week of moderate-intensity aerobic activity spread over 3 or more days [18]. At baseline, 22.3% (191/857) of the 12-month study sample met this criteria and this increased to 36.8% (316/857) at 12 months.

##### Low Medication Adherence

At baseline, 36.3% (311/856) reported low medication adherence. This decreased by 16% to 30.5% (261/856) by 12 months. Those with low adherence had a mean improvement (lower score) of –0.765. Adherent participants worsened by 0.154 (scale 0-4).

##### Hypoglycemic Symptoms

At baseline, 38.8% (329/849) of participants reported 2 or more hypoglycemic symptoms. At 12 months, 30.0% (255/849) reported 2 or more hypoglycemic symptoms. Those with symptoms at baseline had a mean improvement of –1.05. Participants without hypoglycemic symptoms worsened by 0.237 (scale 0-7).

##### Depression

At baseline, 21.3% (182/855) of participants had symptoms of depression, as defined by PHQ-8=10 or more [12]. At 12 months, the number with depression had decreased to 15.6% (133/855). The participants with depression had an improvement of –4.18 in PHQ-8 at 12 months. The participants with lower baseline PHQ-8 had a small improvement of –0.171 in PHQ-8 (scale 0-24).

#### Participants Who Improved in at Least One Condition (Hypothesis 6)

We examined the proportion of all participants (not limited to those with specific conditions) who improved in at least 1 of the 5 criterial conditions. Using effect-size improvements of at least 0.4 as an indication of an improvement, 79.9% of the total 12-month study population (685/857) improved in at least 1 of the 5 criterial variables. About 44% (377/857) improved in 2 or more. The mean number of improvements (out of a possible 5 for those with HbA1c data or 4 for those without) was 1.49 (SD 1.13). The 176 participants who did not improve in any of the 5 criterial variables had a mean of 0.956 criterial conditions, while those who improved in at least one criterion had a mean of 1.72 conditions. There was an r=0.374 correlation (Pearson) between the number of criterial conditions and the number of improvements of 0.4 effect size.

## Discussion

### Principal Findings

Between baseline (preintervention) and 1 year, there were modest, but statistically significant, improvements in 14 of 15 outcome measures, including HbA1c. There were no significant changes in the proportion having cholesterol exams within the preceding year. However, baseline percentages having those tests were high, leaving little room for improvement.

### Healthy People 2020

The Healthy People 2020 [17] goals were to (1) reduce by 10% the proportion of persons with diabetes with an HbA1c value greater than 9.0%; (2) increase the proportion of the diabetic population with an HbA1c value less than 7% HbA1c by 10%; (3) increase the proportion of adults with diabetes who have at least an annual foot examination by 10% from a current 68% to 74.8%; (4) increase the proportion having annual eye exams by 10%; (5) increase the proportion having urinary microalbumin (kidney) tests by 10%; and (6) increase the proportion of adults with diabetes who receive formal diabetes education. By design, the BCBH-D workshops add to the proportion receiving diabetes education. For 4 of 5 goals, the participants in the program exceeded the desired increase of 10%. For the fifth goal (eye exams), the participants in the study were very close to achieving the goal (a mean increase of 9.2%). A year after the intervention, the participants exceeded many of Healthy People 2020 goals, such as improvements in HbA1c and percentage having recommended examinations. In addition, if the findings of this study can be replicated in other settings, the interventions may lead to improvement in both HEDIS and Medicare Star Quality Measures.

### Limitations

The real-world nature of this pragmatic study necessitated the lack of a control group. Thus, we cannot be certain that the improvements observed are not due to other factors or might have occurred during the same time to nonparticipants. Alternative explanations for the improvements might include new medications and health plan initiatives that became available during the period of the study. It is also possible that there were interactions between workshop participation and members’ likelihood of taking advantage of Anthem initiatives and new medications. While these other factors may be important, the consistency of the statistically significant improvements across multiple domains suggests a positive impact of workshop participation.

Attrition may also have affected outcomes (people with negative results may be more likely to avoid 12-month follow-up questionnaires). However, considering the large initial sample (n=1229) and large number of outcomes, there were only a few baseline statistical differences between 12-month completers and noncompleters, suggesting only minimal possible attrition bias.

For the analyses of changes for those with the 4 criteria conditions, regression to the mean might have contributed to the outcomes. However, the large differences in positive change scores for the worse-off compared with the smaller changes for the complementary subset of those relatively better-off suggest that not all of the improvements could have resulted from regression to the mean.

Even with the real-world pragmatic nature of the study, the participants were largely a self-selected population, perhaps more motivated than people who would not enroll. In 2016, the CMS Center for Innovation published a report from a 3000 plus person sample of people receiving Medicare [19]. Among other questions, they asked the likelihood of attending a wellness program if offered in their community in the next 6 months. Nearly 57% said that they were very likely (12.3%), likely (13.9%), or somewhat likely (30.6%) to enroll, and about half of these (46.4%) said they were interested in chronic diseases self-management programs.

### Implications

As a community-based intervention, BCBH-D offered in 2 modes was associated with small but significant nonreinforced benefits, which were sustained for at least 1 year. The 2 different modes were able to reach somewhat different populations. This illustrates that offering more than 1 delivery mode reaches a broader population. Overall, there was little difference in 12-month improvements—participants in both delivery modes benefited similarly.

The program also appeared to meet some Healthy People 2020 diabetes objectives and could contribute to improved HEDIS and Medicare Star ratings. Most importantly, it was associated with clinically significant benefits for those with high HbA1c, for those with depression and hypoglycemia, as well as medication nonadherers and nonexercisers. The benefits differed by individual, but a large majority of the population had meaningful improvements in at least one of the areas. This study demonstrates that the peer-facilitated BCBH-D program, offered outside of the traditional health care system, can assist a national sample of health-plan members in improving their diabetes management, with benefits persisting for at least 1 year. This, and similar multidelivery-mode programs have the potential for making an impact on our growing diabetes population.

## Abbreviations

BCBH-D: Better Choices, Better Health-Diabetes
CLIA: Clinical Laboratory Improvement Amendments
HEDIS: Healthcare Effectiveness Data and Information Set
PHQ-8: Patient Health Questioinaire
